# *In Vitro* Activity of New β-Lactam–β-Lactamase Inhibitor Combinations and Comparators against Clinical Isolates of Gram-Negative Bacilli: Results from the China Antimicrobial Surveillance Network (CHINET) in 2019

**DOI:** 10.1128/spectrum.01854-22

**Published:** 2022-07-12

**Authors:** Yan Guo, Renru Han, Bo Jiang, Li Ding, Fengzhen Yang, Beijia Zheng, Yang Yang, Shi Wu, Dandan Yin, Demei Zhu, Fupin Hu

**Affiliations:** a Institute of Antibiotics, Huashan Hospital, Fudan University, Shanghai, China; b Key Laboratory of Clinical Pharmacology of Antibiotics, Ministry of Health, Shanghai, China; c First Affiliated Hospital of Kunming Medical University, Yunnan, China; d Yantai Yuhuangding Hospital, Shandong, China; e Taizhou Central Hospital, Zhejiang, China; Johns Hopkins University School of Medicine

**Keywords:** β-lactam–β-lactamase inhibitor combinations, difficult-to-treat resistance, cefepime-zidebactam, ceftazidime-avibactam, carbapenemase

## Abstract

Novel β-lactam–β-lactamase inhibitor combinations (BLBLIs) are in clinical development for the treatment of infections caused by carbapenem-resistant and difficult-to-treat resistant (DTR) (defined as resistance to all tested β-lactams and fluoroquinolones) Gram-negative bacilli. This study evaluated the *in vitro* activities of cefepime-zidebactam, ceftazidime-avibactam, cefepime-tazobactam, ceftolozane-tazobactam, and other comparators against 4,042 nonduplicate Gram-negative clinical isolates collected from different regions of China (46 hospitals) in 2019. Based on the pharmacokinetic-pharmacodynamic (PK-PD) breakpoints, cefepime-zidebactam inhibited 98.5% of *Enterobacterales* and 98.9% of Pseudomonas aeruginosa isolates, respectively. Against carbapenem-resistant and difficult-to-treat resistant Gram-negative bacilli, cefepime-zidebactam demonstrated better activity against *Enterobacterales* (96% and 97.2%, respectively) and P. aeruginosa (98.2% and 96.9%, respectively). Among the 379 carbapenem-resistant *Enterobacterales* isolates, the most common carbapenemase genes detected were *bla*_KPC-2_ (64.1%) and *bla*_NDM_ (30.9%). Cefepime-zidebactam showed an MIC_90_ of ≤2 mg/L for 98.8% of *bla*_KPC_-positive isolates and 89.7% of *bla*_NDM_-positive isolates. Ceftazidime-avibactam also showed efficient *in vitro* activity against *Enterobacterales* (93.6%) and P. aeruginosa (87.7%). Ceftazidime-avibactam was active against 97.5% of *bla*_KPC_-positive isolates and 100% of *bla*_OXA-232_-positive isolates. Cefepime-zidebactam inhibited 97.3% of Acinetobacter baumannii isolates with an MIC_50/90_ of 16/32 mg/L. Our study systematically evaluated the *in vitro* activities of these new BLBLIs against a variety of Gram-negative bacilli, provided preclinical data for the approval of these BLBLIs in China, and supported cefepime-zidebactam and ceftazidime-avibactam as potential efficient therapies for infections caused by carbapenem-resistant *Enterobacterales* (CRE), carbapenem-resistant P. aeruginosa (CRPA), and DTR isolates.

**IMPORTANCE** *Enterobacterales*, Pseudomonas aeruginosa, and Acinetobacter baumannii are the most common Gram-negative bacilli to cause nosocomial infections throughout the world. Due to their large public health and societal implications, carbapenem-resistant A. baumannii (CRAB), carbapenem-resistant P. aeruginosa (CRPA), and carbapenem-resistant and third-generation-cephalosporin-resistant *Enterobacteriaceae* were regarded by the World Health Organization (WHO) as a global priority for investment in new drugs in 2017. The present study showed the potent *in vitro* activity of these novel BLBLIs and other comparators against Gram-negative bacillus isolates, including carbapenem-resistant or difficult-to-treat resistant phenotypes. Polymyxins, tigecycline, and ceftazidime-avibactam (except for *bla*_NDM_-positive isolates) were available for the treatment of infections caused by CRE isolates. Currently, cefepime-zidebactam and other BLBLIs have not yet been approved for use in China. Here, our study aimed to evaluate the *in vitro* activities of BLBLIs against Gram-negative bacillus isolates, especially CRE, before clinical use.

## INTRODUCTION

Gram-negative bacilli are causative pathogens in many infections, including pneumonia, bloodstream infections, wound or surgical site infections, and meningitis, in health care settings, which have become a significant public health threat globally (1–3). Results from the China Antimicrobial Surveillance Network (CHINET) (www.chinets.com) for 2021 showed that more than 25% of Klebsiella pneumoniae, 20% of Pseudomonas aeruginosa, and 69% of Acinetobacter baumannii isolates are resistant to imipenem and meropenem. Carbapenem-resistant Gram-negative bacilli have rapidly increased worldwide in the last decade, which is related to the emergence and prevalence of plasmid-mediated extended-spectrum β-lactamases (ESBLs), AmpC cephalosporinases, and carbapenemases among these isolates, conferring resistance to β-lactam antibiotics, and make difficulties in empirical treatment for clinicians (3, 4). Recently, the difficult-to-treat resistant (DTR) phenotype, defined as resistance to all tested β-lactams and fluoroquinolones, has caught attention as it is associated with clinical therapeutic options and patient outcomes. Antimicrobial resistance in these bacteria has significant potential impacts on antibiotic use and patient outcomes (1). Currently, aminoglycosides, polymyxins (colistin and polymyxin B), and tigecycline are the antibiotics available for the treatment of infections caused by these intractable isolates in China but are problematic in their clinical efficacy, their safety profile, and emerging resistance (3, 5–7). New therapeutic development is urgently needed to combat these intractable pathogens. To date, several new β-lactam–β-lactamase inhibitor combinations (BLBLIs) in different stages of development, including ceftazidime-avibactam, ceftolozane-tazobactam, cefepime-zidebactam, meropenem-vaborbactam, and imipenem-relebactam, inhibit class A and class C β-lactamases, and some are active against class B and class D β-lactamases (3, 8, 9). In this study, based on data from the CHINET Antimicrobial Surveillance Network, we evaluated the *in vitro* activity of these newly developed BLBLIs against Gram-negative bacilli and strengthened the epidemiological surveillance of resistance of Gram-negative bacilli to confront an emerging global epidemic.

## RESULTS

### Strain characteristics.

The results of antimicrobial susceptibility testing indicated that 61% of Escherichia coli, 51% of K. pneumoniae, and 44.5% of Proteus mirabilis isolates were resistant to ceftriaxone.

Among the tested *Enterobacterales* isolates, 379/2,656 (14.3%) were carbapenem-resistant *Enterobacterales* (CRE), including K. pneumoniae (74.1%; 281/379), E. coli (10.6%; 40/379), and Enterobacter cloacae (4.5%; 17/379).

For glucose-nonfermenting bacteria, 228/756 (30.2%) and 471/630 (74.8%) were carbapenem-resistant P. aeruginosa (CRPA) and carbapenem-resistant A. baumannii (CRAB), respectively, and 11.9% (316/2,656) of *Enterobacterales* isolates and 8.6% (65/756) of P. aeruginosa isolates were difficult-to-treat resistant (DTR) isolates.

### Susceptibility of Gram-negative bacilli.

The *in vitro* activities of cefepime-zidebactam, ceftazidime-avibactam, cefepime-tazobactam, ceftolozane-tazobactam, and other comparator agents against 4,042 clinical isolates are summarized in Tables 1 to 3. Cefepime-zidebactam exhibited potent antibacterial activity against all *Enterobacterales* isolates (*n* = 2,656) with an MIC_50/90_ of 0.06/1 mg/L. A total of 98.5% of isolates were inhibited at the provisional cefepime-zidebactam pharmacokinetic-pharmacodynamic (PK-PD) breakpoint (≤8 mg/L), with 24 E. coli, 4 K. pneumoniae, 7 Proteus rettgeri, 3 P. mirabilis, 1 E. cloacae, and 1 Serratia marcescens isolates showing MICs of ≥16 mg/L among the various genera of *Enterobacterales*. Besides cefepime-zidebactam, ceftazidime-avibactam was also active against all *Enterobacterales* clinical isolates with an MIC_50/90_ of 0.25/4 mg/L. Among 171 ceftazidime-avibactam-resistant isolates, cefepime-zidebactam showed an MIC of 8 mg/L or lower against 84.1% of the tested isolates (data not shown). Apart from cefepime-zidebactam and ceftazidime-avibactam, tigecycline (96.5% susceptible) and amikacin (90.4% susceptible) also displayed potent activity against *Enterobacterales*. The rate of susceptibility to cefepime-tazobactam was 85.8%, similar to those for polymyxin B (81.4% susceptible) and meropenem (85.6% susceptible), which showed good activity against the tested isolates. More than 60% of the *Enterobacterales* isolates were susceptible to ceftolozane-tazobactam (74.2% susceptible), imipenem (74.6% susceptible), piperacillin-tazobactam (75.5% susceptible), cefoperazone-sulbactam (69.8% susceptible), and ceftazidime (60.5% susceptible). The following other comparator agents showed limited activity: cefepime (55.3% susceptible), ceftriaxone (46.1% susceptible), aztreonam (54.7% susceptible), ciprofloxacin (42.1% susceptible), levofloxacin (48.1% susceptible), and trimethoprim-sulfamethoxazole (53.7% susceptible) (Table 1).

**TABLE 1:** *In vitro* activities of cefepime-zidebactam and comparator agents against 2,656 *Enterobacterales* isolates^*a*^

Antibacterial agent	*Enterobacterales* (*n* = 2,656)					CRE (*n* = 379)				DTR *Enterobacterales* (*n* = 316)			
	MIC range (mg/L)	MIC_50_ (mg/L)	MIC_90_ (mg/L)	% R	% S	MIC_50_ (mg/L)	MIC_90_ (mg/L)	% R	% S	MIC_50_ (mg/L)	MIC_90_ (mg/L)	% R	% S
Cefepime-zidebactam	≤0.03 to >64	0.06	1	NA^*b*^	98.5^*c*^	1	4	NA	96^*c*^	1	4	NA	97.2^*c*^
Ceftazidime-avibactam	≤0.03 to >64	0.25	4	6.4	93.6	4	>64	34.8	65.2	2	>64	25.3	74.7
Cefepime-tazobactam	≤0.03 to >64	0.06	64	NA	85.8^*d*^	>64	>64	NA	10.8^*d*^	>64	>64	NA	6.6^*d*^
Ceftolozane-tazobactam	≤0.06 to >128	0.5	128	23.9	74.2	128	>128	98.9	1.1	128	>128	100	0
Tigecycline	≤0.06 to >32	0.25	2	0.8	96.5	0.5	2	0.8	95.5	1	2	0.9	95.9
Polymyxin B^*e*^	≤0.125 to >16	0.5	>16	18.6	81.4	0.5	2	9.5	90.5	0.5	1	7.6	92.4
Imipenem	≤0.06 to >128	0.5	32	18	74.6	64	128	98.4	0.8	64	128	99.4	0
Meropenem	≤0.03 to >64	≤0.03	64	14	85.6	>64	>64	98.4	0.8	>64	>64	99.7	0
Piperacillin-tazobactam	1 to >256	4	>256	19.6	75.5	>256	>256	96.8	2.1	>256	>256	100	0
Cefoperazone-sulbactam	≤1 to >128	4	>128	21.7	69.8	>128	>128	98.7	1.1	>128	>128	100	0
Cefepime	≤0.06 to >128	0.5	>128	36.6	55.3	>128	>128	97.1	1.1	>128	>128	99.4	0
Ceftazidime	≤0.25 to >32	1	>32	34.2	60.5	>32	>32	98.2	1.1	>32	>32	99.4	0
Ceftriaxone	≤0.5 to >32	4	>32	52.1	46.1	>32	>32	99.7	0.3	>32	>32	100	0
Cefuroxime	≤0.25 to >32	>32	>32	62.9	33.5	>32	>32	99.5	0.5	>32	>32	100	0
Cefazolin	≤0.5 to >32	>32	>32	72	20.1	>32	>32	99.7	0.3	>32	>32	100	0
Amikacin	0.5 to >128	2	16	9.3	90.4	64	>128	50.7	49.3	>128	>128	59.5	40.5
Aztreonam	≤1 to >128	2	>128	41.5	54.7	>128	>128	91.8	7.7	>128	>128	99.7	0
Ciprofloxacin	≤0.06 to >8	1	>8	50.4	42.1	>8	>8	90.5	7.7	>8	>8	100	0
Levofloxacin	≤0.125 to >16	1	>16	43.5	48.1	>16	>16	87.1	9.5	>16	>16	98.1	0
Trimethoprim-sulfamethoxazole	≤0.25 to >32	1	>32	46.3	53.7	>32	>32	60.4	39.6	>32	>32	60.4	39.6

a CRE, carbapenem-resistant *Enterobacterales*; DTR, difficult-to-treat resistant; % R, percentage of resistant isolates; % S, percentage of susceptible isolates.

b NA, not available.

c Cefepime-zidebactam MICs were interpreted using a provisional breakpoint of ≤8 mg/L based on the PK-PD breakpoint.

d Cefepime-tazobactam MICs were interpreted using a provisional breakpoint of ≤16 mg/L based on the PK-PD breakpoint.

e Polymyxin B MICs were interpreted using the EUCAST breakpoint of colistin (≤2 mg/L, susceptible; ≥2 mg/L, resistant).

**TABLE 2:** *In vitro* activities of cefepime-zidebactam and comparator agents against 756 P. aeruginosa isolates^*a*^

Antibacterial agent	P. aeruginosa (*n* = 756)					CRPA (*n* = 228)				DTR P. aeruginosa (*n* = 65)			
	MIC range (mg/L)	MIC_50_ (mg/L)	MIC_90_ (mg/L)	% R	% S	MIC_50_ (mg/L)	MIC_90_ (mg/L)	% R	% S	MIC_50_ (mg/L)	MIC_90_ (mg/L)	% R	% S
Cefepime-zidebactam	≤0.03 to >64	2	8	NA^*b*^	98.9^*c*^	4	8	NA	98.2^*c*^	8	16	NA	96.9^*c*^
Ceftazidime-avibactam	≤0.03 to >64	2	16	12.3	87.7	4	64	32	68	16	>64	66.2	33.8
Cefepime-tazobactam	≤0.03 to >64	4	32	NA	87.7^*d*^	16	64	NA	68.4^*d*^	32	>64	NA	23.1^*d*^
Ceftolozane-tazobactam	≤0.06 to >128	1	4	7.1	90.2	2	>128	18	76.3	8	>128	38.5	49.2
Polymyxin B^*e*^	0.25 to >16	1	2	4.4	95.6	1	1	3.1	96.9	1	1	1.5	98.5
Imipenem	0.125 to >128	2	32	29.4	59.4	16	64	97.4	0.9	32	>128	100	0
Meropenem	≤0.03 to >64	0.5	16	18.5	75.3	8	64	61.4	24.6	32	>64	93.8	0
Piperacillin-tazobactam	≤2 to >256	8	256	16.7	67.3	32	>256	37.3	37.3	256	>256	87.7	0
Cefoperazone-sulbactam	≤1 to >128	8	64	19.8	65.9	32	>128	44.3	33.8	128	>128	92.3	0
Cefepime	≤0.06 to >128	4	32	14.2	76.9	16	128	35.5	49.6	32	>128	83.1	0
Ceftazidime	≤0.25 to >32	4	>32	22.1	71.2	16	>32	44.3	42.5	>32	>32	92.3	0
Amikacin	≤1 to >128	4	8	3.8	95.4	4	32	9.6	89	8	>128	26.2	70.8
Aztreonam	≤1 to >128	8	64	32.7	56.1	32	128	53.9	33.3	64	>128	90.8	0
Ciprofloxacin	≤0.06 to >8	0.25	8	22	68.5	1	>8	37.3	47.8	>8	>8	76.9	0
Levofloxacin	≤0.125 to >16	1	16	28.4	61.8	2	>16	49.6	38.2	16	>16	95.4	0

a CRPA, carbapenem-resistant P. aeruginosa; DTR, difficult-to-treat resistant.

b NA, not available.

c Cefepime-zidebactam MICs were interpreted using a provisional breakpoint of ≤32 mg/L based on the PK-PD breakpoint.

d Cefepime-tazobactam MICs were interpreted using a provisional breakpoint of ≤16 mg/L based on the PK-PD breakpoint.

e Polymyxin B MICs were interpreted using the EUCAST breakpoint of colistin (≤2 mg/L, susceptible; ≥2 mg/L, resistant).

**TABLE 3:** *In vitro* activities of cefepime-zidebactam and comparator agents against 630 A. baumannii isolates^*a*^

Antibacterial agent	A. baumannii (*n* = 630)					CRAB (*n* = 471)			
	MIC range (mg/L)	MIC_50_ (mg/L)	MIC_90_ (mg/L)	% R	% S	MIC_50_ (mg/L)	MIC_90_ (mg/L)	% R	% S
Cefepime-zidebactam	≤0.03 to >64	16	32	NA^*b*^	97.3^*c*^	16	64	NA	96.6^*c*^
Cefepime-tazobactam	≤0.03 to >64	64	>64	NA	30.6^*d*^	64	>64	NA	7.9^*d*^
Tigecycline	≤0.06 to >32	1	4	4	89.5	1	4	3.4	88.5
Polymyxin B^*e*^	≤0.125 to >16	0.5	1	3.2	96.8	0.5	0.5	3.4	96.6
Imipenem	≤0.06 to >128	64	128	74.4	25.2	64	128	99.6	0.2
Meropenem	≤0.03 to >64	64	>64	74.3	25.4	64	>64	99.4	0.6
Cefepime	≤0.06 to >128	64	>128	74	24.4	128	>128	97.2	1.7
Ceftazidime	≤0.25 to >32	>32	>32	74.4	24	>32	>32	96.8	3.2
Ceftriaxone	≤0.5 to >32	>32	>32	75.4	13.5	>32	>32	96.8	0.8
Piperacillin-tazobactam	≤2 to >256	>256	>256	74.1	24	>256	>256	96.8	2.5
Cefoperazone-sulbactam	≤1 to >128	64	>128	67.6	26.3	128	>128	89.2	4
Amikacin	≤1 to >128	>128	>128	61.7	38.3	>128	>128	81.3	18.7
Ciprofloxacin	≤0.06 to >8	>8	>8	74.4	24.8	>8	>8	96.2	3.2
Levofloxacin	≤0.125 to >16	8	>16	63.8	25.7	16	>16	82.6	4.2
Trimethoprim-sulfamethoxazole	≤0.25 to >32	32	>32	60.8	39.2	>32	>32	76.9	23.1

a CRAB, carbapenem-resistant A. baumannii; DTR, difficult-to-treat resistant.

b NA, not available.

c Cefepime-zidebactam MICs were interpreted using a provisional breakpoint of ≤64 mg/L based on the PK-PD breakpoint.

d Cefepime-tazobactam MICs were interpreted using a provisional breakpoint of ≤16 mg/L based on the PK-PD breakpoint of P. aeruginosa.

e Polymyxin B MICs were interpreted using the EUCAST breakpoint of colistin (≤2 mg/L, susceptible; ≥2 mg/L, resistant).

A total of 756 clinical isolates of P. aeruginosa were highly inhibited by cefepime-zidebactam with an MIC_50/90_ of 2/8 mg/L at a PK-PD breakpoint of ≤32 mg/L (98.9% susceptible). The rate of susceptibility of P. aeruginosa to cefepime-zidebactam was similar to or slightly higher than those for ceftazidime-avibactam (87.7% susceptible), ceftolozane-tazobactam (90.2% susceptible), polymyxin B (95.6% susceptible), and amikacin (95.4% susceptible) (Table 2). The rates of susceptibility to many commonly used broad-spectrum β-lactams, i.e., cefepime-tazobactam, cefepime, ceftazidime, and meropenem, of P. aeruginosa ranged from 70% to 80%, and those for other comparator agents, i.e., imipenem, old BLBLIs, aztreonam, and fluoroquinolones, ranged from 50% to 70%.

The MIC_50/90_ value of cefepime-zidebactam against 630 A. baumannii isolates was 16/32 mg/L (Table 3). Among the tested isolates, 97.3% were susceptible to cefepime-zidebactam based on ≤64 mg/L. Polymyxin B and tigecycline were the available agents showing excellent activity against A. baumannii isolates, with susceptibilities of 89.5% and 96.8%, respectively. The rates of susceptibility to amikacin and trimethoprim-sulfamethoxazole were around 40%. These isolates were highly resistant to other β-lactams, with or without BLBLIs, as well as the fluoroquinolones tested, with susceptibility rates of less than 30%.

### Susceptibility of carbapenem-resistant organisms.

Overall, the CRE isolates were inhibited by cefepime-zidebactam with an MIC_50/90_ of 1/4 mg/L at ≤8 mg/L. Cefepime-zidebactam retained good activity with an MIC_90_ in the range of 0.125 to 16 mg/L against *bla*_KPC_-positive (*n* = 243), *bla*_NDM_-positive (*n* = 117), *bla*_IMP_-positive (*n* = 8), *bla*_OXA-232_-positive (*n* = 7), *bla*_VIM_-positive (*n* = 1), as well as carbapenemase-negative (*n* = 3) isolates (Table 4). The MIC_90_ value of ceftazidime-avibactam was lower than the susceptibility breakpoint, with 97.5% and 100% susceptible *bla*_KPC_-positive and *bla*_OXA-232_-positive isolates, respectively. Tigecycline and polymyxin B showed good *in vitro* activity against CRE, with susceptibilities of 95.5% and 90.5%, respectively. The rates of susceptibility to amikacin and trimethoprim-sulfamethoxazole of the CRE isolates were 49.3% and 39.6%, respectively.

**TABLE 4:** *In vitro* activities of cefepime-zidebactam and comparator agents against isolates of carbapenem-resistant *Enterobacterales* carrying carbapenemase genes

Group (no. of isolates)	Cefepime-zidebactam^*a*^			Ceftazidime-avibactam			Cefepime-tazobactam^*b*^			Ceftolozane-tazobactam			Tigecycline			Polymyxin B^*c*^		
	MIC_50_ (mg/L)	MIC_90_ (mg/L)	% S	MIC_50_ (mg/L)	MIC_90_ (mg/L)	% S	MIC_50_ (mg/L)	MIC_90_ (mg/L)	% S	MIC_50_ (mg/L)	MIC_90_ (mg/L)	% S	MIC_50_ (mg/L)	MIC_90_ (mg/L)	% S	MIC_50_ (mg/L)	MIC_90_ (mg/L)	% S
MBL^*d*^ positive																		
NDM (117)	0.25	16	89.7	>64	>64	0.9	>64	>64	6.8	>128	>128	1.7	0.5	2	96.6	0.5	8	88.9
IMP (8)	0.125	0.5	100	>64	>64	12.5	16	>64	50	>128	>128	12.5	0.25	8	87.5	0.5	8	87.5
VIM (1)	0.125	0.125	100	32	32	0	4	4	100	>128	>128	0	0.25	0.25	100	0.5	0.5	100

MBL negative, serine carbapenemase positive																		
KPC (243)	1	2	98.8	2	4	97.5	64	>64	10.7	128	>128	0	1	2	95.5	0.5	1	91.8
OXA-232 (7)	1	2	100	1	2	100	64	>64	100	128	>128	0	2	2	100	0.5	>16	85.7

MBL negative, serine carbapenemase negative (3)	4	8	100	32	>64	33.3	64	>64	100	>128	>128	33.3	1	4	66.7	0.5	>16	66.7

a Cefepime-zidebactam MICs were interpreted using a provisional breakpoint of ≤8 mg/L based on the PK-PD breakpoint.

b Cefepime-tazobactam MICs were interpreted using a provisional breakpoint of ≤16 mg/L based on the PK-PD breakpoint.

c Polymyxin B MICs were interpreted using the EUCAST breakpoint of colistin (≤2 mg/L, susceptible; ≥2 mg/L, resistant).

d MBL, metallo-β-lactamase.

Moreover, the rate of susceptibility to cefepime-zidebactam of CRPA was higher than those for amikacin (98.2% versus 89%) and polymyxin B (98.2% versus 96.9%), whereas the rates of susceptibility were 76.3% for ceftolozane-tazobactam and 68% for ceftazidime-avibactam as the most active comparators. Except for imipenem and meropenem, CRPA isolates were moderately resistant to other β-lactams, aztreonam, and fluoroquinolones, with susceptibility rates of 30% to 50%.

For CRAB, cefepime-zidebactam, tigecycline, and polymyxin B showed high susceptibility rates of 96.6%, 88.5%, and 96.6%, respectively, and amikacin and trimethoprim-sulfamethoxazole showed limited activity, with susceptibility rates of 18.7% and 23.1%, respectively. The MICs of other agents were higher, with MIC_90_ values of >32 mg/L.

### Susceptibility of DTR isolates.

Cefepime-zidebactam inhibited 97.2% of DTR *Enterobacterales* isolates with an MIC_50/90_ of 1/4 mg/L at ≤8 mg/L, and 74.7% of DTR *Enterobacterales* isolates were susceptible to ceftazidime-avibactam with an MIC_50/90_ of 2/>64 mg/L. Only tigecycline (95.9% susceptible) and polymyxin B (92.4% susceptible) displayed greater *in vitro* activity than cefepime-zidebactam and ceftazidime-avibactam against all DTR *Enterobacterales* isolates (Table 1).

A total of 96.9% of DTR P. aeruginosa isolates were susceptible to cefepime-zidebactam with an MIC_50/90_ of 8/16 mg/L at ≤32 mg/L. The rates of susceptibility to amikacin, ceftolozane-tazobactam, and ceftazidime-avibactam of DTR P. aeruginosa isolates were 70.8%, 49.2%, and 33.8%, respectively. Only polymyxin B (98.5% susceptible) demonstrated greater *in vitro* activity than the above-described agents against DTR P. aeruginosa isolates (Table 2).

### Detection of carbapenemase genes.

In this study, 99.2% (376/379) of the CRE isolates had a single carbapenemase gene, and only 3 isolates were negative for all five common carbapenemase genes (Table 4). Among these carbapenemase genes, 64.1% (243/379) of isolates were *bla*_KPC-2_ positive, 18.5% (70/379) were *bla*_NDM-5_ positive, 12.4% (47/379) were *bla*_NDM-1_ positive, 2.1% (8/379) were *bla*_IMP_ positive, 1.8% (7/379) were *bla*_OXA-232_ positive, and 0.3% (1/379) were *bla*_VIM_ positive, respectively. Additionally, *bla*_KPC-2_ was mainly detected in K. pneumoniae (80.1%; 225/281), S. marcescens (90.9%; 10/11), Citrobacter freundii (44.4%; 4/9), and Morganella morganii (100%; 1/1). The highest prevalences of *bla*_NDM-5_ were 82.5% (33/40) in E. coli and 55.6% (5/9) in Klebsiella aerogenes isolates. *bla*_NDM-1_ was the predominant type of carbapenemase gene among E. cloacae (70.6%; 12/17) and P. rettgeri (100%; 9/9) isolates.

## DISCUSSION

Of particular concern is the spread of antimicrobial-resistant Gram-negative bacillus isolates, especially CRE, P. aeruginosa, and A. baumannii, which has substantially increased morbidity and mortality rates and caused nosocomial outbreaks (10, 11). The emergence of antimicrobial resistance continues to outpace the development of new agents (12). Novel BLBLIs such as ceftazidime-avibactam and ceftolozane-tazobactam significantly reduce the disease burden for patients and improve serious adverse outcomes against Gram-negative bacilli as effective treatment options. Surveillance of resistance to these novel BLBLIs has been continuously performed in the Chinese mainland since 2017, although they were not approved by the National Medical Products Administration.

In this study, 98.5% of *Enterobacterales* and 98.9% of P. aeruginosa isolates were inhibited by cefepime-zidebactam based on PK-PD breakpoints of ≤8 mg/L and ≤32 mg/L (13), respectively. In a lab of International Health Management Associates (IHMA) study (12), the authors observed that cefepime-zidebactam inhibited 98.5% of *Enterobacterales* and 59.6% of P. aeruginosa isolates. There are currently no Clinical and Laboratory Standards Institute (CLSI), European Committee on Antimicrobial Susceptibility Testing (EUCAST), or U.S. Food and Drug Administration (FDA) clinical breakpoints of cefepime-zidebactam, so according to its PK-PD breakpoint (≤32 mg/L), P. aeruginosa had a rate of susceptibility to cefepime-zidebactam of 99.6%, whereas it was 98.9% in our study. The potent activity of cefepime-zidebactam against CRE, P. aeruginosa, and A. baumannii isolates harboring carbapenemase genes has been previously reported. In another study of a worldwide surveillance program, Sader et al. (14) reported that 99.3% of CRE isolates (*n* = 153) had cefepime-zidebactam MICs of ≤8 mg/L, similar to the results of this study (98.5%).

The DTR phenotype, a novel category in the study of Gram-negative bacteremia, focuses on treatment-limiting resistance to all first-line agents. The DTR phenotype was defined as an isolate that tests not susceptible (intermediate or resistant) to all β-lactam categories, including carbapenems and fluoroquinolones, and it was demonstrated that isolates that were not susceptible to first-line agents were associated with increased patient mortality and clinical failure. Karlowsky et al. (12) studied 13,248 Gram-negative clinical isolates at 26 U.S. hospitals from 2015 to 2017 for the SMART global surveillance program and found that overall, 1% of infections exhibited DTR. Specific DTR rates observed in that study were 0.3% for E. coli, 0.6% to 1.0% for Enterobacter spp., 0.6% to 3.0% for Klebsiella spp., and 8.4% for P. aeruginosa (data not shown). In our study, we observed slightly higher DTR rates of 1.2% for E. coli, 0.04% to 0.5% for Enterobacter spp., 9.3% for Klebsiella spp., and 8.6% for P. aeruginosa. The differences in DTR rates between the 2 studies may reflect the characteristics of the strains among different regions and different specimen sources. Kadri et al. reported that mortality was significantly higher for DTR than for carbapenem-resistant, extended-spectrum-cephalosporin-resistant, or fluoroquinolone-resistant infections (15). The *in vitro* effect was also observed in our CRE as well as P. aeruginosa isolates. But cefepime-zidebactam still showed good activity against these carbapenem-resistant organisms (CROs) (96% to 98.2%) and DTR isolates (96.9% to 97.2%).

In this study, more than 89.5% of the CRE and CRAB isolates tested were susceptible to tigecycline and polymyxin B. Additionally, 96.9% of CRPA isolates were susceptible to polymyxin B. Ceftazidime-avibactam has been used for the treatment of infections caused by *bla*_KPC_- or *bla*_OXA-48_-positive isolates. *bla*_KPC_-positive isolates showed a low rate of resistance to ceftazidime-avibactam (2.5%), but the majority (87.5% to 100%) of *bla*_NDM_-positive isolates were resistant to ceftazidime-avibactam. The major resistance mechanisms that confer reduced susceptibility to ceftazidime-avibactam are as follows: the production of metallo-β-lactamases (MBLs) such as NDM, VIM, or IMP; *bla*_KPC_ variants; and the transposition of KPC with porin deficiency (3, 16). During the clinical use of ceftazidime-avibactam, several researchers have observed a change from the KPC-2 to the KPC-33 carbapenemase of CRE isolates but lower MICs of carbapenems (often restoring susceptibility to imipenem and low-level resistance to meropenem) because the KPC variants exhibiting single-amino-acid substitutions in their Ω-loop (positions 164 to 179, particularly the Asp179Tyr substitution) and two additional regions (one close to the hinge loop at positions 240 to 243 and one covering positions 263 to 277) lead to an enhanced affinity for ceftazidime and reduced binding to avibactam (16–19). Similar to avibactam, zidebactam lacks direct β-lactamase-inhibitory activity against MBLs. But cefepime-zidebactam exhibited potent activity against MBL-producing isolates, contingent on zidebactam’s unique penicillin binding protein 2 (PBP2) binding action (20). Due to high-affinity Gram-negative bacterial PBP2 binding, zidebactam demonstrates antibacterial activity against various *Enterobacteriaceae* and P. aeruginosa isolates (14).

There were limitations to our study. First, some new agents that also show potent activity, such as meropenem-vaborbactam, imipenem-relebactam, and cefiderocol, have not been evaluated at this time due to difficulties in the ordering process. Second, a homology analysis of resistant isolates, especially CRO isolates, has not been carried out to clarify the characteristics of their spread in China.

### Conclusion.

We studied a recent nationwide collection of Gram-negative bacilli and observed that new BLBLIs, especially cefepime-zidebactam and ceftazidime-avibactam, demonstrated potent *in vitro* activity against *Enterobacterales* (susceptibility rates of 98.5% and 93.6%, respectively) and P. aeruginosa (98.9% and 87.7%, respectively) isolates producing important β-lactamases, including MBLs (except for ceftazidime-avibactam), KPCs, and OXA-232, for which treatment agents are limited. The results from this study support the use of cefepime-zidebactam and ceftazidime-avibactam as potential therapies for infections caused by CRE, CRPA, and DTR isolates.

## MATERIALS AND METHODS

### Compliance with ethical standards.

The study protocol was approved by the Institutional Review Board of Huashan Hospital, Fudan University (no. 2019-460).

### Clinical isolates.

The China Antimicrobial Surveillance Network (CHINET) is a multicenter bacterial resistance surveillance program in operation since 2005 in China. In 2019, 46 hospitals in 28 provinces or cities collected up to 4,042 nonduplicate, clinically significant Gram-negative isolates from CHINET, including Klebsiella pneumoniae (*n* = 979), Escherichia coli (*n* = 900), P. aeruginosa (*n* = 756), A. baumannii (*n* = 630), Enterobacter cloacae (*n* = 172), Proteus mirabilis (*n* = 119), Serratia marcescens (*n* = 118), K. aerogenes (*n* = 103), Morganella morganii (*n* = 89), Citrobacter freundii (*n* = 84), Proteus vulgaris (*n* = 51), Proteus rettgeri (*n* = 29), and Klebsiella oxytoca (*n* = 12). Among the tested clinical isolates, 23.6% of the isolates were isolated from patients in the intensive care unit, followed by outpatient and emergency departments (18.5%), urology surgery (6.7%), respiratory medicine (5.6%), neurosurgery departments (4.2%), and other departments. A total of 33.9% of the tested isolates were isolated from sputum, followed by urine (22.5%), blood (12.1%,), secreta (7.7%), bronchoalveolar lavage fluid (3.9%), pus (2.8%), wound (2.7%), abdominal fluid (2.0%), bile (1.7%), shunt fluid (1.3%), drain (1.2%), and other sources (8.2%). Species identification was performed at each participating site and confirmed by the central laboratory using matrix-assisted laser desorption ionization–time of flight mass spectrometry (Vitek MS; bioMérieux, France). Quality control was performed according to Clinical and Laboratory Standards Institute (CLSI) guidelines using E. coli ATCC 25922 and ATCC 35218, K. pneumoniae ATCC 700603, and P. aeruginosa ATCC 27853 for antimicrobial susceptibility testing.

### Antimicrobial susceptibility testing.

MICs were determined by the reference broth microdilution method recommended by the CLSI. Cefepime-zidebactam, ceftazidime-avibactam, cefepime-tazobactam, ceftolozane-tazobactam, and other comparator agents were tested using a dried customized commercially prepared microdilution panel (Sensititre; Thermo Fisher Scientific) in the study. Quality control and test results were interpreted according to 2021 CLSI breakpoints (21) for all agents tested except for cefepime-zidebactam, tigecycline, and polymyxin B, for which CLSI criteria were not available. Tigecycline MICs were interpreted using the U.S. Food and Drug Administration (FDA) MIC breakpoints for *Enterobacterales* (22). Cefepime-zidebactam MICs were interpreted using provisional breakpoints based on anticipated clinical data (13, 23) (≤8 mg/L for *Enterobacterales*, ≤32 mg/L for P. aeruginosa, and ≤64 mg/L for A. baumannii). Cefepime-tazobactam MICs were interpreted using provisional breakpoints of ≤16 mg/L for *Enterobacterales* and P. aeruginosa based on PK-PD studies (24). Polymyxin B was explained by European Committee on Antimicrobial Susceptibility Testing (EUCAST) MIC interpretative breakpoints of colistin (25).

In this study, isolates with meropenem or imipenem resistance phenotypes were considered carbapenem-resistant organisms (CROs). Difficult-to-treat resistance phenotypes were defined by testing resistance to all tested β-lactams (including carbapenems and β-lactamase inhibitor combinations) and fluoroquinolones (15).

### Detection of carbapenemase genes.

Carbapenem-resistant *Enterobacterales* (CRE) isolates were selected for analysis of carbapenemase. The five most common carbapenemase genes (*bla*_KPC_, *bla*_NDM_, *bla*_IMP_, *bla*_VIM_, and *bla*_OXA-48_) were confirmed for all of the CRE isolates by PCR with specific primers and DNA sequencing, as described previously (26).
